# NBPF9 Gene May Be Involved in Congenital Hypopituitarism: A Whole-Genome Study of a Boy with Pituitary Stalk Interruption Syndrome and His Family

**DOI:** 10.1155/2020/5401738

**Published:** 2020-07-18

**Authors:** Cheng-Zhi Wang, Ling-Ling Guo, Qing-Hua Guo, Yi-Ming Mu

**Affiliations:** ^1^Department of Endocrinology, The First Medical Center of PLA General Hospital, Beijing 100853, China; ^2^Department of Endocrinology, Sun Yat-Sen Memorial Hospital, Sun Yat-Sen University, Guangzhou 510120, China; ^3^Department of Endocrinology, Beijing Electric Teaching Hospital of Capital Medical University, Beijing, 100073, China; ^4^Department of Endocrinology, Hainan Branch of Chinese PLA General Hospital, Sanya, Hainan 572000, China

## Abstract

Pituitary stalk interruption syndrome (PSIS) is a rare congenital defect manifesting as various degrees of anterior pituitary hormone deficiency. Scattered familial cases have been found, revealing some genetic variants. However, most of the previous research studies involved an affected sibling, and the gene spectra of the patients' entire family have rarely been reported. We conducted a study of a family consisting of a PSIS patient with his unaffected sibling and healthy parents of Han Chinese background using whole-genome sequencing. Bioinformatic analysis was carried out, and mutations related to PSIS, single-nucleotide variants (SNVs), insertion-deletion (InDELs), and structural variations (SVs) in all the four samples were filtered. After Sanger sequencing, we confirmed the variants obtained and selected three candidate genes for functional verification. The gene variations in this boy with PSIS and his lineal relatives are reported herein; *de novo* sequencing revealed that the *NBPF9* gene may be involved in the pathogenesis of PSIS.

## 1. Introduction

Pituitary stalk interruption syndrome (PSIS) is a rare congenital defect with an estimated incidence of 0.5/100,000 births. PSIS causes various symptoms of pituitary hormone deficiency, including growth retardation and infertility [1–3].

Previous studies have revealed that genetic changes may contribute to its etiology. Studies on patients born to consanguineous parents have revealed germline mutations in some genes that may be responsible for the disease. For example, Fernandez-Rodriguez et al. reported a homozygous mutation, 301-302delAG, in the *PROP1* gene in two consanguineous sisters [4]. Reynaud et al. identified a novel *PROKR2* variant (p.Ala51Thr) in two familial cases [1]. In addition, Tatsi et al. [5] found a novel heterozygous nonsense mutation (c.799C > T, p.Q267X) in the *TGIF* gene in a female PSIS patient with a single central incisor; her father had the same mutation but was asymptomatic.

However, the above studies only provide partial details of the genetic variants responsible, as the samples were limited to siblings or one parent. In our study, we examined DNA from a PSIS boy, his unaffected parents, and his sister. We hope that our findings help in obtaining more information about this rare disease. Moreover, we performed functional verification of a novel gene found by sequencing to explain the pathogenesis in-depth.

## 2. Materials and Methods

### 2.1. Participants

**Figure 1:**
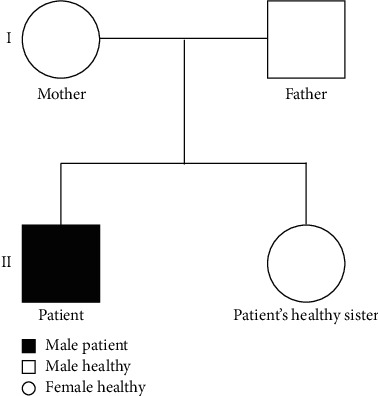
Pedigree of this family.

A boy with PSIS was referred to Chinese PLA General Hospital; all his family members, including his unaffected sister and healthy parents, were also included. The study was approved by the Ethics Committee of the General Hospital of Chinese PLA, and informed consent was obtained from all participants prior to their participation in the study. The family lineage is displayed in Figure 1.

### 2.2. Whole-Genome Sequencing and Analysis

Genomic DNA was extracted from peripheral blood mononuclear cells (PBMCs) from the family members in our discovery cohort. Exome capturing was performed to collect whole exons of human genomic DNA. The exon-enriched DNA libraries were sequenced in 100 bp paired-end reads using a HiSeq2000 sequencer (Illumina, San Diego, CA); coverage of 100x was typically achieved for each sample.

Qualified DNA was randomly fragmented using a Covaris Ultrasonic Processor. DNA libraries were constructed through end repair, addition of A to tails, purification, and PCR amplification. The pair-end libraries were sequenced using an Illumina HiSeq X Ten machine.

### 2.3. Genome Sequencing and Analysis

After verification, the different libraries were sequenced based on the effective concentration and required raw data by Illumina HiSeq X Ten sequencing platform after being verified. Clean reads were obtained after removing adaptor sequences and low mapping quality reads. Read alignment to the reference genome hg38 was performed with Burrows–Wheeler Aligner MEM (BWA-MEM) (0.7.15). Duplicated reads were marked and removed by Picard (2.5.0). The alignment results were sorted using SAMtools (0.1.18). Recalibration of base quality scores and local realignment around InDELs were performed using Genome Analysis Toolkit (GATK) (3.6) [6]. SNVs and InDELs for each sample were determined using GATK HaplotypeCaller. Structural variants (SVs) were evaluated via LUMPY [7]. The database annotation, analysis, and global overview of variation were performed by ANNOVAR [8] and Variant Effect Predictor [9]. The genes *SOX3*, *PROP1*, *LHX3*, *LHX4*, *HESX1*, *OTX2*, *PROKR2*, *TGIF*, *GPR161*, and *CDON* have been analyzed in gene variation sets [1, 2, 10–13].

### 2.4. Sanger Sequencing

Candidate variants were further confirmed by Sanger sequencing executed by Taihegene Biotechnology (Beijing). Purified PCR amplification products were obtained after amplification of target gene fragments in DNA samples using a Biometra PCR instrument. Chromas 2 software was used for manual interpretation, and sequences were obtained from NCBI GenBank (http://www.ncbi.nlm.nih.gov/sites/entrez/). The first-generation sequencing results were compared with the second-generation sequencing results, and genes were screened for functional verification. The primer sequences are shown in Table 1.

### 2.5. Functional Verification

#### 2.5.1. Cell Culture and Transfection

Human embryonic stem cell (HESCS) H1 cells were cultured in mTeSR1 medium in pretreated culture plates covered with Matrigel (BD Company). The cells were passaged at approximately 70% confluence. siRNAs targeting *MUC12*, *NBPF9*, and *TYW1B* were designed by Gemma Company. Transfection was based on optimization methods reported in domestic and foreign literature [14–16]. For further experiments, RNA was extracted at 24 h after transfection and protein at 48 h after transfection.

#### 2.5.2. Western Blotting

Protein samples were boiled (5 min, 95°C), separated by SDS-PAGE, and transferred onto polyvinylidene difluoride membranes. The membranes were blocked for 1 h in 5% bovine serum albumin (BSA) in TBST (20 mM Tris-HCl, pH 7.6, 150 mM NaCl, and 0.1% Tween 20). Primary antibodies against LHX3 and PITX1 (Enogene Biotech, Nanjing, China) were diluted in TBST containing 5% BSA and then applied to the membranes overnight at 4°C at a dilution of 1 : 1,000. After being washed three times with TBST, the membranes were incubated with horseradish peroxidase-conjugated secondary antibodies for 1 h. The membranes were washed three times with TBST and developed with Chemiluminescence ECL Plus-Western Blotting detection reagents (Amersham Biosciences, Piscataway, NJ, USA).

## 3. Results

### 3.1. Clinical Features

The boy with PSIS was referred to our hospital due to growth retardation. He was born in a nonconsanguineous family, and none of his relatives had a history of growth retardation or short stature. He was born via breech delivery with a body weight of 3,200 g and a height of 50 cm. After he was 5 years old, his parents noticed growth retardation compared with other children of his age. When he was then admitted to a local children's hospital, his height was 100 cm (−2.15 SDS), and he showed a delayed bone age of 3. According to GH stimulation tests, the maximum value detected at the time was 0.69 ng/mL, along with a low IGF-1 level estimated at 24.45 ng/mL. Pituitary CT revealed a hypoplastic pituitary gland. He was diagnosed with GH deficiency, and hormone replacement therapy was initiated.

In 2013, when the boy was 8 years old, he was admitted to Chinese PLA General Hospital; his height was 106 cm (−1.07 SDS), and he had a body weight of 16.5 kg. He had a single central incisor, sharp palate, and single transverse palmar crease; he could not easily bend the fourth and fifth fingers of both hands. In addition to GH deficiency, he was diagnosed with hypothyroidism (free T4: 8.35 pmol/mL (reference interval: 12–22 pmol/mL); TSH: 6.54 mU/L (reference interval: 0.35–5.5 mU/L)) and hypoadrenalism (F 8 am: 128.6 nmol/L (reference interval: 198.7–797.5 nmol/L at 24 h UFC) at admission. His bone age was that of a 5-year-old boy. The patient was treated with GH, hydrocortisone, and levothyroxine thereafter. Records from the latest follow-up in 2015 showed a height of 131 cm (−1.06 SDS) and a bone age of approximately 8 years.

**Figure 2:**
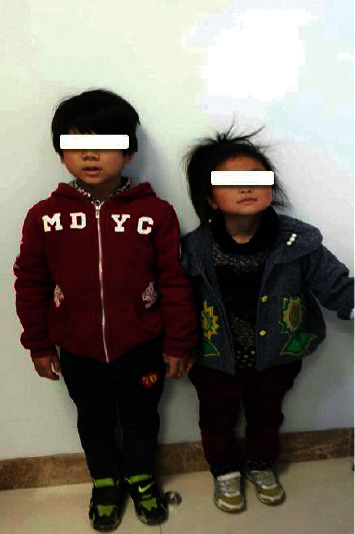
The PSIS boy and his healthy sibling. The left is the PSIS boy (8 years old), and his healthy sister (5 years old) was on the right.

His younger sister, who was the second child in this family, exhibited normal growth with no deformities. As a 5-year-old girl, her height was 111.5 cm and her weight was 18 kg, which were in the normal range for her age. The results of her hormone laboratory tests were normal. The boy's parents denied consanguineous marriage or family histories of similar manifestations. The mother confirmed that she did not receive radiation or take any drugs during pregnancy.  Figure 2 shows the appearance of the PSIS boy and his healthy sister.

### 3.2. Molecular and *In Silico* Findings

#### 3.2.1. Variant Statistics

*(1) SNVs and InDELs*. We obtained 4,935,685 SNVs from the four samples as well as 9.644 InDELs; based on genetics, genomic region, and functional annotation, we filtered SNV results for each sample. 48.1% were intron variants and 37.7% were intergenic variants. In exon region, 52.3% were synonymous variants.

*(2) Structural Variation (SV)*. LUMPY and the DGV database were used to assess all SVs in the 4 samples, SVs were detected in different regions of the genomic region, and 97.06% SVs were located in intergenic region.

#### 3.2.2. Analysis of Reported Genes

Mutations in the genes *SOX3, PROP1*, *LHX3*, *LHX4*, *HESX1*, *OTX2*, *PROKR2*, *TGIF*, *GPR161*, and *CDON* have been reported. The current database is not related to PSIS mutation locus information matching, according to the variation function for filtering exonic regions of the mutation loci. There are five genes that showed regional variation, including *LHX4*, *PROP1*, *CDON*, *TGIF1*, and *PROKR2*, as shown in Table 2.

### 3.3. De Novo Mutation Analysis

We aimed to find mutations that manifested as hybrid-type genome mutation loci in the PSIS boy but not genomic mutations in his parents. A total of 19 specific mutations were found (Table 3).

### 3.4. Inheritance Pattern Analysis

According to the pedigree information of the family, the variation and variation annotation information for each individual were used for grade selection; mutation loci were filtered by grade and function (nonsynonymous mutations) to obtain 267 candidate mutation loci. The 267 variation sites are located in 207 genes. Further analysis was performed by dbSNP, ClinVar, Exome Sequencing Project, 1000 Genomes Project, and Exome Aggregation Consortium database.  Table 4 presents 11 filtered recessive genetic variations.

As most of the above genes have rarely been studied, we selected *NBPF9*, *MUC12*, and *TYW1B* as candidate genes to further analyze their roles in nervous system development and cancer [17, 18].

### 3.5. Sanger Sequencing

**Figure 3:**
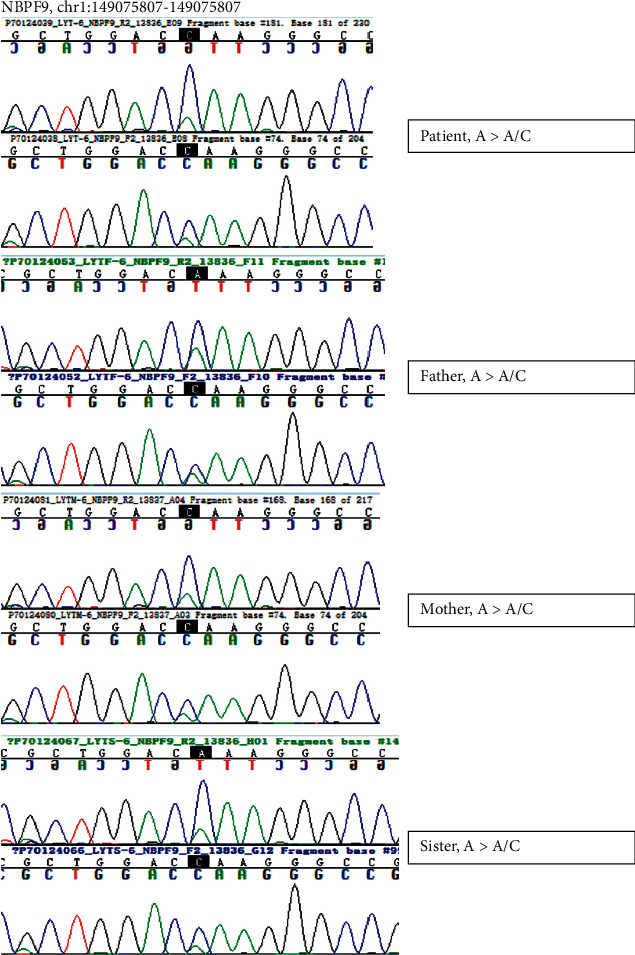
Mutations in the *NBPF9* gene and Sanger sequencing validation.

We further confirmed the candidate variations by Sanger sequencing, and the results are depicted in Figure 3.

### 3.6. Protein Structure Prediction

**Figure 4:**
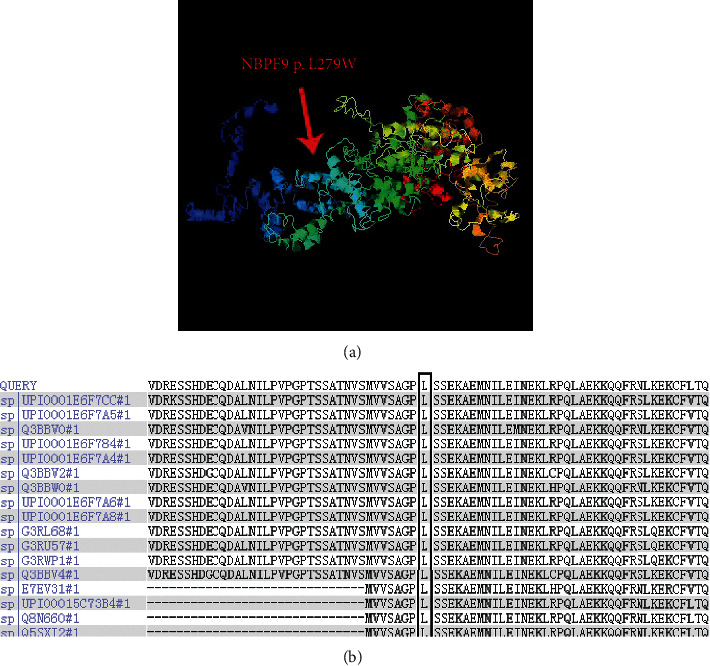
Structure of the protein encoded by the *NBPF9* gene. (a). Structure of the protein encoded by the *NBPF9* gene, which would cause protein structure variation. (b). Conserved sequence in the region of the NBPF9 p.L279W mutation.

After protein structure prediction, it was found that the NBPF9 p.L279W point mutation would cause protein structure variation. The conserved amino acid sequence is illustrated in Figure 4. This sequence shows good conservation.

### 3.7. Cell Culture and Western Blotting

**Figure 5:**
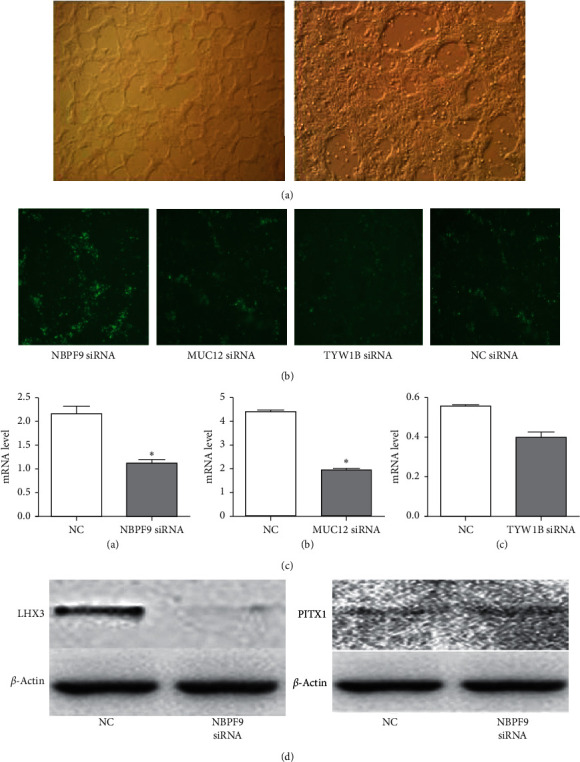
Culture status of human embryonic stem cells, siRNA transfection effect, and target gene expression. (a) Human embryonic stem cells on the left showed scattered growth and human embryonic stem cells on the right gradually formed cell colonies. (b) Cells after transfection under a fluorescence microscope. (c) siRNA transfection after 24 h, with efficiency above 60%. qPCR results after silencing the expression of each gene are shown below to verify the inhibitory effect. (d) Expression of LHX3 and PITX1 by western blotting, in which LHX3 was significantly decreased after treatment with siRNA targeting *NBPF9*.

Figure 5 shows the cell culture of HESCS (H1 cells). siRNAs targeting the candidate genes *NBPF9*, *MUC12*, and *TYW1B* were transfected into cells, and inhibition rates of more than 50% were obtained with the NBPF9 and MUC12 groups. At the protein level, LHX3 abundance decreased after transfection with *NBPF9* siRNA. For PITX1, no significant change was observed between the *NBPF9* transfection group and the control group.

## 4. Discussion

As a rare and complicated defect, PSIS is thought to be caused by genetic changes. However, genetic variants are found in only 5% of cases. Sporadic cases have been collected and studied, but little information was found. Through familial cases, we obtained some reported mutations, but in these studies, only the father or mother's DNA was examined. Here, we evaluate a family with a PSIS boy and a healthy sibling and parents, which may reveal more details of the etiology of PSIS.

We analyzed the reported genes *LHX4*, *PROP1*, *CDON*, *TGIF*, and *PROKR2* in our study. LHX4/LHX3 are members of the LIM-homeobox family and regulate the proliferation and differentiation of pituitary lineages [1, 2, 12]. PROP1 is involved in early patterning, proliferation, positional determination, and terminal differentiation of the pituitary gland [4, 13]. CDON is a component of a cell-surface receptor complex that mediates cell-cell interactions between muscle precursor cells [11]. Tatsi reported TGIF as an active transcriptional corepressor of *SMAD2* that may participate in the transmission of nuclear signals during development and in adults [5]. PROKR2 is thought to be involved in endocrine angiogenesis and neuronal migration and is expressed in the hypothalamus and pituitary gland [1]. However, none of the mutation frequencies were significant. In other words, the mutation frequencies were equal to those of the healthy group. We further performed inheritance pattern analysis and found 11 genes with related variations but that have rarely been studied; thus, little is known about their functions.

In our previous study related to sporadic cases, we proposed that compromised genetic interactions among multiple signaling pathways are likely to underlie the pathogenesis of sporadic PSIS [19]. Therefore, multigenetic causes may explain the pathogenesis of PSIS. Therefore, in this study, we attempted to identify novel gene variations. After bioinformatics analysis, we selected candidate variants for further research.

NBPF9 belongs to the neuroblastoma breakpoint family (NBPF) family, the members of which are characterized by binding to the repetitive DUF1220 protein domain. Gene copy number variation exists in the region of human chromosome 1q21. 1. DUF1220 domains have been associated with many diseases related to development and neurogenesis, such as small head deformity, autism and schizophrenia, mental retardation, congenital heart disease, neuroblastoma, and congenital kidney and urinary tract abnormalities, and previous studies have reported that DUF1220 is associated with lung cancer [17].

We found that the level of LHX3 decreased after transfection with *NBPF9* siRNA. The *LHX3* and *PITX1* genes appear to play important roles during pituitary development. LHX3 is a LIM homologous domain transcription factor that is one of the earliest pituitary transcription factors expressed in development; it determines the specific differentiation of tissues and cells in vertebrates and invertebrates by regulating the expression of some genes. Currently, three types of *LHX3* autosomal recessive inheritance have been identified in humans, and all patients have multiple pituitary hormone deficiencies [21–23]. *PITX1*, a member of the homologous domain protein transcription factor (PITX) family, was found in fruit flies in 1996; the encoded protein activates the transcription of opioid melanocortin precursor and is involved in the derivation and development of the pituitary gland [24]. The above research results indicate that LHX3 plays an important role in the development of the pituitary; in contrast, the *NBPF9* gene has only been shown to be an important link in pituitary development.

In our study, we describe the landscape of the gene spectrum of a PSIS family and, based on further experiments, speculate that the *NBPF9* gene is likely a regulatory factor affecting the development of the pituitary. Nonetheless, more samples of entire PSIS families are needed to explain the exact pathogenesis.

## 5. Conclusions

The findings of this study provide considerable information about the gene spectrum of PSIS as a rare disease. However, there is a lack of evidence regarding all of the reported genes. More samples are needed to assess clinical applicability. The *NBPF9* gene may be involved in congenital hypopituitarism [20].

## Figures and Tables

**Table 1:** Primer sequences.

Gene	Primer sequence (5′-3′)
FCGBP	F: 5′-AACgAggggTgAggTTCTTAC3′ R: 5′-CCACCACAAAACATCCTTTTCA-3″
RANGAP1	F: 5′-gAgACAACCAAAggAAgCCTgT-3′ R: 5′-CAgAgACATgAAggAggggAgT-3′
FAM155A	F: 5′-CAggCCTggAATTTgCTTTTAT-3′ R: 5′-gTTgCAgTAAggCAAgAgATCg-3′
EPHA6	F: 5′-ATCTCAggACAgTTCCTATTC-3′ R: 5′-TTTTTCTgCAgCCCCggCTgTg-3′
AGAP9	F: 5′-TgCTCTACCAAgTCTggTCTgC-3′ R: 5′-ATgTCATggAAAAAgTCgAgCA-3″
NBPF9	F: 5′-CACTTgTACCCAggAgCCAgAg-3′ R: 5′-TggggACTAgACATCCCAgTCT-3′
TYW1B	F: 5′-gCTCTCCTTgTTgAATTgAT-3′ R: 5′-TgCATCAACTAATgggTgAAA-3′
MUC12	F: 5′-ACCAAgCCTCAgTgAgAAATC-3′ R: 5′-ggACAgTgCTgTgTCAgTTg-3′

**Table 2:** Analysis of reported genes.

Pos	Gene	Chr	Start	End	Ref	Alt	Type
Exonic	LHX4	1	180230592	180230592	T	C	Synonymous SNV
Exonic	LHX4	1	180274389	180274389	A	G	Nonsynonymous SNV
Exonic	PROP1	5	177995875	177995875	T	C	Nonsynonymous SNV
Exonic	PROP1	5	177995907	177995907	A	G	Synonymous SNV
Exonic	CDON	11	125961075	125961075	A	T	Nonsynonymous SNV
Exonic	CDON	11	125978366	125978366	C	T	Synonymous SNV
Exonic	CDON	11	126021374	126021374	C	T	Nonsynonymous SNV
Exonic	TGIF	18	3457541	3457541	A	G	Synonymous SNV
Exonic	TGIF1	18	3457609	3457609	C	T	Nonsynonymous SNV
Exonic	PROKR2	20	5302073	5302073	G	T	Synonymous SNV
Exonic	PROKR2	20	5302085	5302085	G	A	Synonymous SNV
Exonic	PROKR2	20	5302204	5302204	C	T	Nonsynonymous SNV
Exonic	PROKR2	20	5302610	5302610	C	G	Synonymous SNV
Exonic	PROKR2	20	5302730	5302730	G	A	Synonymous SNV

**Table 3:** Specific mutations in the PSIS boy.

Pos	Gene	Chr	Start	End	Ref	Alt	Type
Exonic	TRIOBP	chr22	37724147	37724147	G	A	Nonsynonymous SNV
Exonic	GP1BA	chr17	4933823	4933900	AGC……CCC	—	Nonframeshift deletion
Exonic	FCGBP	chr19	39902043	39902043	A	G	Nonsynonymous SNV
Exonic	RANGAP1	chr22	41254465	41254467	TCC	—	Nonframeshift deletion
Exonic	POTEG	chr14	19433854	19433854	C	T	Nonsynonymous SNV
Exonic	DNAJB6	chr7	157385630	157385630	G	A	Nonsynonymous SNV
Exonic	FAM155A	chr13	107866364	107866365	TG	—	Frameshift deletion
Exonic	FAM155A	chr13	107866367	107866379	CGC……GCT	—	Frameshift deletion
Exonic	GAGE2A,	chrx	49590729	49590729	C	T	Nonsynonymous SNV
	GAGE2B,						
	GAGE2C						
Exonic	EPHA6	chr3	96814795	96814797	GAG	—	Nonframeshift deletion
Exonic	FCGBP	chr19	39902109	39902109	T	G	Nonsynonymous SNV
Exonic	TAL1	chr1	47219924	47219925	AG	—	Frameshift deletion
Exonic	FLG	chr1	152312347	152312347	A	G	Nonsynonymous SNV
Exonic	MUC12	chr7	100997864	100997864	G	A	Nonsynonymous SNV
Exonic	RASA4B	chr7	102485138	102485138	C	T	Nonsynonymous SNV
Exonic	AGAP9	chr10	47502991	47502991	C	T	Nonsynonymous SNV
Exonic	ZNF705E	chr11	71818808	71818808	T	C	Nonsynonymous SNV
Exonic	NPIPB11	chr16	29383805	29383805	C	T	Nonsynonymous SNV
Exonic	ZNF705E	chr11	71818809	71818809	G	A	Stop-gain

**Table 4:** Inheritance pattern analysis.

Gene	Chr	Start	End	Ref	Alt	Type
*SPATA31A3*	chr9	66987043	66987043	G	T	Nonsynonymous SNV
*OR9I1*	chr11	58118963	58118963	G	T	Nonsynonymous SNV
*ABCB11*	chr2	168944621	168944621	G	A	Nonsynonymous SNV
*AGAP9*	chr10	47503470	47503470	G	A	Nonsynonymous SNV
*TYW1B*	chr7	72728896	72728896	C	T	Stop-gain
*AGAP9*	chr10	47502579	47502579	C	T	Nonsynonymous SNV
*AGAP9*	chr10	47502650	47502650	G	C	Nonsynonymous SNV
*NBPF9*	chr1	149075807	149075807	A	C	Nonsynonymous SNV
*AGAP9*	chr10	47502343	47502343	T	C	Nonsynonymous SNV
*AGAP9*	chr10	47502604	47502604	A	G	Nonsynonymous SNV
*ZNF180*	chr19	44497294	44497294	A	G	Nonsynonymous SNV

## Data Availability

The data used to support the findings of this study are available from the corresponding author upon reasonable request.

## Abbreviations

PSIS: Pituitary stalk interruption syndrome
MRI: MR imaging
SNV: Single-nucleotide variants
InDEL: Insertion-deletion
SV: Structural variation
WES: Whole-exome sequencing
NGS: Next-generation sequencing.
